# Commentary on the use of the reproduction number *R* during the COVID-19 pandemic

**DOI:** 10.1177/09622802211037079

**Published:** 2021-09-27

**Authors:** Carolin Vegvari, Sam Abbott, Frank Ball, Ellen Brooks-Pollock, Robert Challen, Benjamin S Collyer, Ciara Dangerfield, Julia R Gog, Katelyn M Gostic, Jane M Heffernan, T Déirdre Hollingsworth, Valerie Isham, Eben Kenah, Denis Mollison, Jasmina Panovska-Griffiths, Lorenzo Pellis, Michael G Roberts, Gianpaolo Scalia Tomba, Robin N Thompson, Pieter Trapman

**Affiliations:** 1 Medical Research Council Centre for Global Infectious Disease Analysis, Department of Infectious Disease Epidemiology, School of Public Health, 4615 Imperial College London, London, UK; 2 Center for the Mathematical Modelling of Infectious Diseases, 4906 London School of Hygiene & Tropical Medicine, UK; 3 School of Mathematical Sciences, 6123 University of Nottingham, UK; 4 Bristol Veterinary School, 1980 University of Bristol, UK; 5 NIHR Health Protection Research Unit in Behavioural Science and Evaluation at the University of Bristol, UK; 6 EPSRC Centre for Predictive Modelling in Healthcare, 3286 University of Exeter, UK; 7 Somerset NHS Foundation Trust, UK; 8 65899 Isaac Newton Institute for Mathematical Sciences, UK; 9 Department of Applied Mathematics and Theoretical Physics, University of Cambridge, UK; 10 Department of Ecology and Evolution, 2462 University of Chicago, USA; 11 Centre for Disease Modelling, Mathematics & Statistics, 7991 York University, Canada; 12 COVID Modelling Task-Force, The Fields Institute, Canada; 13 Big Data Institute, Li Ka Shing Centre for Health Information and Discovery, 6396 University of Oxford, UK; 14 Department of Statistical Science, 4919 University College London, UK; 15 Division of Biostatistics, College of Public Health, 2647 The Ohio State University, USA; 16 Department of Actuarial Mathematics and Statistics, Heriot-Watt University, UK; 17 The Big Data Institute, Li Ka Shing Centre for Health Information and Discovery, University of Oxford, Oxford, UK; 18 Wolfson Centre for Mathematical Biology, Mathematical Institute and The Queen's College, University of Oxford, Oxford, UK; 19 Department of Mathematics, 5292 The University of Manchester, UK; 20 The Alan Turing Institute, UK; 21 School of Natural and Computational Sciences and New Zealand Institute for Advanced Study, Massey University, New Zealand; 22 Department of Mathematics, 9318 University of Rome Tor Vergata, Italy; 23 Mathematics Institute, 2707 University of Warwick, Coventry, UK; 24 Zeeman Institute for Systems Biology and Infectious Disease Epidemiology Research, 2707 University of Warwick, Coventry, UK; 25 Department of Mathematics, 7675 Stockholm University, Sweden

**Keywords:** Reproduction number, COVID-19 pandemic

## Abstract

Since the beginning of the COVID-19 pandemic, the reproduction number R has become a popular epidemiological metric used to communicate the state of the epidemic. At its most basic, R is defined as the average number of secondary infections caused by one primary infected individual. R seems convenient, because the epidemic is expanding if R>1 and contracting if R<1 . The magnitude of R indicates by how much transmission needs to be reduced to control the epidemic. Using R in a naïve way can cause new problems. The reasons for this are threefold: (1) There is not just one definition of R but many, and the precise definition of R affects both its estimated value and how it should be interpreted. (2) Even with a particular clearly defined R , there may be different statistical methods used to estimate its value, and the choice of method will affect the estimate. (3) The availability and type of data used to estimate R vary, and it is not always clear what data should be included in the estimation. In this review, we discuss when R is useful, when it may be of use but needs to be interpreted with care, and when it may be an inappropriate indicator of the progress of the epidemic. We also argue that careful definition of R , and the data and methods used to estimate it, can make R a more useful metric for future management of the epidemic.

## What is the reproduction number R ?

Since the start of the novel coronavirus (severe acute respiratory syndrome-coronavirus-2 (SARS-CoV-2)) pandemic, the reproduction number R has become a popular summary statistic, used by policymakers to assess the state of the epidemic and the efficacy of interventions, and by the media to communicate the progress of the epidemic to the general public. The primary appeal of R is that it offers a single number that indicates whether the transmission of the pathogen is increasing or decreasing, depending on whether R is above or below one. Early R estimates for SARS-CoV-2 in different countries were in the range of 2.0–6.5^1,2^. However, the use of R can be problematic in terms of both its definition and estimation. Its usefulness is precisely because it is a summary statistic rather than a basic parameter describing the dynamic processes of infection, transmission and recovery. To understand how R is calculated and how it can be affected by interventions, the epidemic process needs to be considered in more detail. When epidemic numbers are small or concentrated in possibly atypical parts of a population, R may be an unreliable descriptor of the state of the outbreak.

**Figure 1.**
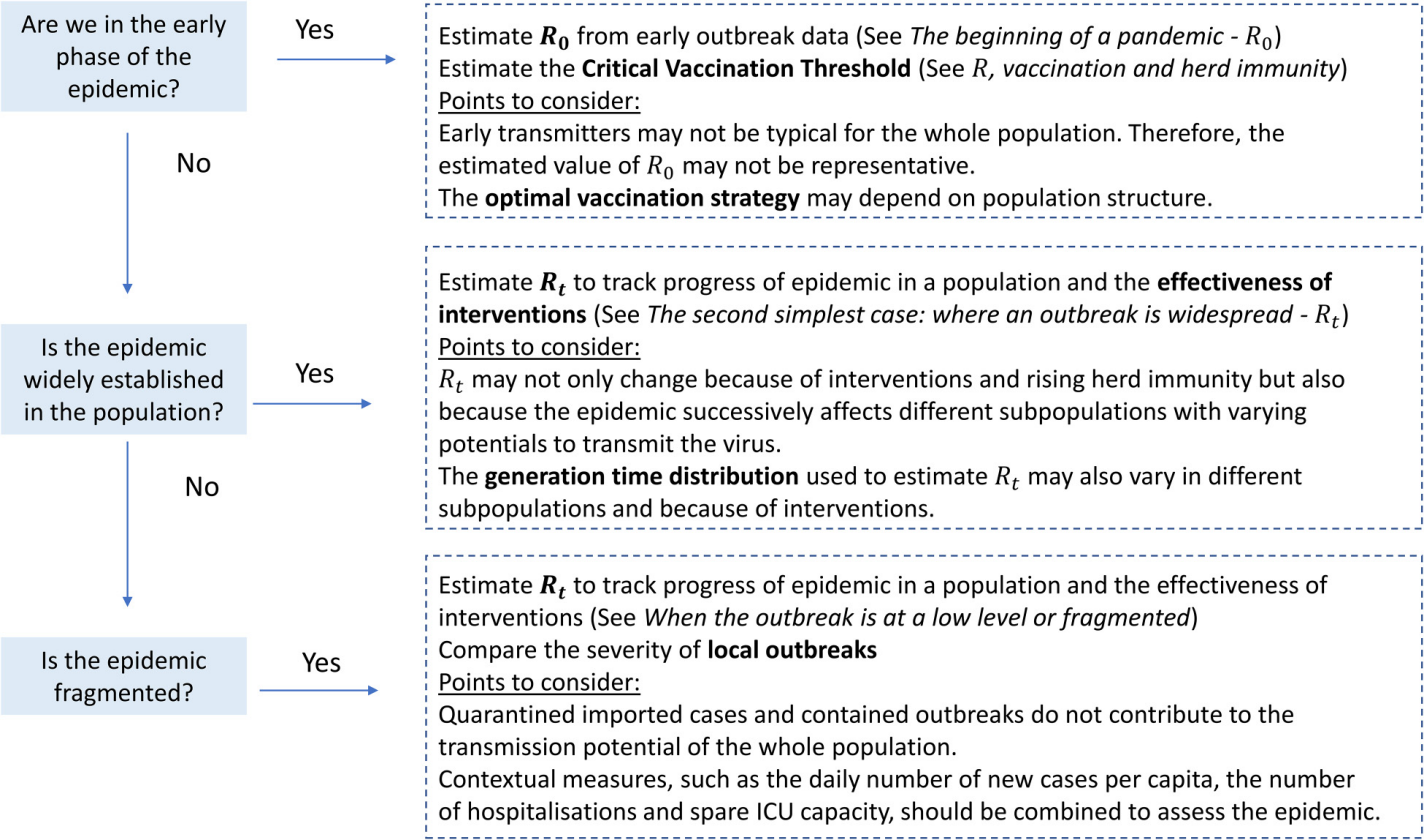
Flowchart summarising the main points explained in the main text depending on the state of the epidemic.

In this paper, we discuss these issues and determine the situations when the reproduction number R is most useful for assessing and communicating the state of an outbreak (see Figure 1). We focus on the definitions of different types of R , for example, the basic reproduction number or the effective reproduction number that can be considered different quantities and their applicability to different phases of an epidemic. However, as we explain below, care must also be taken with subtly different definitions of the same type of R , for example, when using different models to analyse the progress of an epidemic.

### The beginning of a pandemic – $R_{0}$

In the early stages of a new outbreak of an infectious disease, we can define an initial R value, known as the *basic reproduction number* $R_{0}$ , that is the average number of individuals infected by each infectious individual in a fully susceptible population^3–5^. An outbreak resulting from one infected individual may die out within a few infection generations by chance^6,7^. Otherwise, if $R_{0}>1$ , the incidence of cases will grow exponentially, with on average $R_{0}^{n}$ cases in the nth generation. Already, this simple description introduces a number of concepts and assumptions. An individual’s *infection generation* specifies their position in the chain of infections, the (n−1)th generation infects the $n^{\text{th}}$ generation, and so on. It also assumes an underlying scenario (model) in which the average number of susceptibles infected by each infective stays the same over successive infection generations, and ignores the depletion of susceptibles. (We refer to those members of the population who are uninfected and susceptible to infection as *susceptibles*, and those that are infected and infectious as *infectives*.) The potential importance of these assumptions depends on the contact structure of the population, to which we return below.

Thus, $R_{0}$ (and other R values to be defined later) is not just a property of the infectious agent (pathogen). It depends on demography, and whatever human behaviour is associated with the possibility of infectious contact (an *effective contact* is one that results in transmission if made with a susceptible, while a contact in the common sense of the word has a certain probability of transmission). For the simplest models, $R_{0}>1$ implies that an introduction of infection will result in an epidemic. Furthermore, if there were no interventions or changes in behaviour, then the proportion of the population infected during the entire course of an epidemic would be approximately the non-zero solution of the equation $P=1-e^{-R_{0}P}$ (e.g. if $R_{0}=2$ , then P≈0.8 ). This result is referred to as the *final size equation* and underscores the fact that during an epidemic it is not generally true that everybody will be infected at some point.

Individuals may vary considerably in their susceptibility to infection and in their propensity to pass it on through their biology or behaviour. Age is often an important determinant. If the population is grouped in some way, so that for instance some groups have higher R values than others, then the overall outbreak is expected to grow as described by an $R_{0}$ that depends on all of these values, and also depends on how each group infects the others, i.e. on the R values between groups as well as within them ( $R_{0}$ is then the dominant eigenvalue of the matrix of R values^3,5,8^). The first few stages of the outbreak may be atypical, depending on which group is first infected.

For the simplest mathematical model of the beginning of an outbreak, it is assumed that because only a small fraction of the population has been infected, all potential contacts are with susceptibles. This may be an unrealistic assumption because human interaction networks tend to be clustered (e.g. through households, workplaces or schools). Growth through successive generations of infection, which is the basis for defining $R_{0}$ , does not translate simply into time because the *generation interval* of an infection (the time interval back from the instant when a susceptible is infected, to that when their infector was infected) is variable, and infection generations may overlap temporally. Typically, growth in the early stages is faster than the simple assumption of a fixed average generation time would suggest and this is a major problem in estimating $R_{0}$ from early outbreak data. In addition, the implicit assumption is that all infectives are identifiable as such. If there is a significant proportion of asymptomatic cases, an estimate of $R_{0}$ may be affected by the time from when an asymptomatic infective has become infected to when he/she is expected to infect susceptibles. If this timing is the same for asymptomatic and symptomatic cases, then the estimate for $R_{0}$ will be unaffected.

### The second simplest case: where an outbreak is widespread – $R_{t}$

When the pandemic is well-established in a country (or region), with large numbers of cases most of which are internal to the country, an ‘effective reproduction number’ at time t , $R_{t}$ (sometimes denoted $R_{e}$ or $R_{eff}$ ), is a useful descriptor of the progress of the outbreak (Figure 1). Again, the concept is of an average of how many new cases each infectious case causes. The value of $R_{t}$ may be affected by interventions: typically the aim is to reduce $R_{t}$ below one and to as small a value as possible. For models including detailed, and therefore complex, contact networks there may be more than one way of defining $R_{t}$ ; however, definitions should always agree that the value of $R_{t}$ is 1 when the expected number of new infections is constant.

The relevance of the assumptions here (large numbers of cases, mostly internal to the region) is that in such circumstances we expect $R_{t}$ to have a fairly stable value that changes substantially over time only when interventions are introduced or cease. The definition of $R_{t}$ here is in terms of actual new infectious cases, i.e. excluding potentially infectious contacts with individuals who have been infected and are immune to reinfection. As the number of immune individuals grows large compared to the entire population, the spread of infections will gradually slow, because many contacts will be with immune individuals, and hence the value of $R_{t}$ will be reduced. The level of immunity at which $R_{t}=1$ is the *herd immunity threshold* (see the ‘Methods’ section on vaccination and herd immunity below).

### When the outbreak is at a low level or fragmented the concept of R may be less useful

If the outbreak is at a low level either because it has run its course or because of successful interventions, the definition and the use of an R value are problematic (Figure 1). At low levels of prevalence, there will (as in the early stages of the outbreak) be greater statistical variability. Additionally, there are likely to be heterogeneities associated with the infection being unevenly spread among different subgroups of the population (possibly dependent on age, behaviour or geographical location^9^), with some parts of the population having had more exposure than others. There may also be local variability in interventions, and it may not be easy to allow for the effect of some cases being introductions from outside the population under consideration. If the outbreak is fragmented, particularly when close to elimination, it will make more sense to think of it as composed of separate local outbreaks, which can be modelled separately, rather than trying to specify an average R value overall.

### Relating R to details of the infection process

If the population is heterogeneous or structured, defining a reproduction number needs care, as the number of new cases, an infective is expected to cause will depend on both their infectiousness and how well connected they are. It has been shown that in the early stages of an epidemic, when the relevant contact structures of a population are not known and interventions are not targeted, assuming a homogeneous contact structure results in conservative estimates of $R_{0}$ and the required control effort. However, designing targeted intervention strategies requires reliable information on infectious contact structures^10^. There are several basic ways to use structured population models to capture departures from the simplest epidemic models. The four most common are (i) household models, (ii) multi-type models, (iii) network models, and (iv) spatial models.

In a household model, every person in the population is assumed to be part of a single household, which is typically small, and may even be of size one. Those in the same household have a higher probability of infecting each other than is the case for two people chosen randomly from the population. In this model, reproduction numbers can still be defined^11,12^. The most commonly used is the household reproduction number $R_{*}$ , which is the expected number of members of other households that are infected by people from a primary infected household. It is still possible to consider the average number of susceptibles infected by a single infectious person. However, for this to be useful, the average has to be computed in a sophisticated way, because the number of people a person can infect will depend on how many members of the same household are still susceptible when s/he becomes infectious^13^.

A second way of modelling heterogeneity in the population is to assume that the population can be subdivided into groups. The groups may be defined through age bands, social activity levels, health status, type of job, place of residence and so on. Characteristics such as susceptibility, infectivity and frequency of contact may depend on an individual’s group, but all those in a single group have the same characteristics. It is often assumed that all these groups are large. If there are regular inter-group contacts then the largest eigenvalue of the so-called next-generation matrix^5,8^ has many similar properties to those of $R_{0}$ for an epidemic spreading in a homogeneously mixing population, although the final size equation is generally not satisfied.

A third way of introducing heterogeneity is to represent the population by a network, where transmission is only possible between people sharing a link in the network. For many network models, it is still possible to define a reproduction number^14^. It is important to note that the person initially infected in a population is often atypical and should be ignored in computing or estimating the reproduction number. A useful extension is a mixture of a network model and a homogeneous mixing model, in which both regular and casual contacts are captured. In this extension, a reproduction number with the desired threshold properties can be defined^15^.

Sometimes most transmission is restricted to people living close to each other, and spatial models are useful when physical location should be incorporated. For these, it is often difficult to define a reproduction number because there is no phase in which the number of infected is growing exponentially^16,17^. If standard estimation methods are used where there is a considerable spatial component then the estimates will be close to one, even when the spread is highly supercritical and transmission needs to be much reduced to control the epidemic.

## R , vaccination and herd immunity

As immunity builds up in a population through infection during the course of an epidemic, even when the contact rate between individuals remains the same (assuming no change in interventions), both the chance that a contact is susceptible to infection and the effective reproduction number, $R_{t}$ , will decrease. Herd immunity is achieved when enough individuals have become immune so that $R_{t}$ falls below the value 1 without the need to reduce contacts among individuals by non-pharmaceutical interventions.

Vaccination provides another means of building up immunity in a population. Depending on the coverage, it can slow or halt the spread of an epidemic, preventing individual infection or limiting experiences of the disease. All vaccination programmes aim to achieve sufficient immunity in the population that $R_{t}<1$ without modifying contact patterns among individuals. In this situation, there are insufficient susceptibles in the population for sustained transmission. The susceptible proportion of a population for which $R_{t}=1$ is known as the critical vaccination threshold (CVT). When the susceptible proportion is below this threshold, there is herd immunity, which means that the population is protected from a major outbreak even though not everyone is vaccinated or otherwise immune.

In simple mathematical models (e.g. models in which the population is only subdivided into susceptible, infected and recovered individuals), the CVT is determined by the basic reproduction number $R_{0}$ . Specifically, vaccination of a uniform randomly chosen proportion $1-(1/R_{0})$ of the population is sufficient to create herd immunity and prevent an epidemic, as long as the vaccine-induced immunity is sufficiently long-lasting^18^. As a simple example, if $R_{0}=2$ then 50% of a population would need to be vaccinated or otherwise immune to prevent outbreaks. If $R_{0}=3$ , as is approximately the case for COVID-19, then 67% of a population would need to be vaccinated or immune. When setting such vaccination targets, waning immunity needs to be taken into account. The implementation and impact of a vaccination programme depend on whether vaccination is performed before or during an outbreak^19,20^.

As outlined above, the population structure affects the reproduction numbers $R_{0}$ and $R_{t}$ as well as the probability that an epidemic will spread. Therefore, it has important effects on the threshold for herd immunity and the optimal vaccination strategy. For models with small mixing groups such as households, the basic reproduction number $R_{0}$ , as defined in the ‘The beginning of a pandemic – $R_{0}$ ’ section, does not provide a good indicator of whether or not an epidemic can take off because repeated contacts within households are likely even in the early stages of an outbreak. However, in the early stages of an epidemic, between-household contacts are likely to be with individuals in otherwise fully susceptible households, so the reproduction number $R_{*}$ , which is given by the average number of between-household contacts that emanate from a typical within-household epidemic^21,22^ can be used instead. For household models, herd immunity is achieved if a uniform randomly chosen proportion $1-(1/R_{*})$ of all *households* in a population is fully vaccinated.

For COVID-19, a toy model has been used to illustrate the effect of population heterogeneity on herd immunity. It showed^23^ that age structure and variation in social contacts among individuals could reduce the herd immunity threshold to 43%, almost a third less than that for a homogeneous population. Assuming a more extreme variation in social contact rates and that the most exposed individuals become infected first, another study estimates that the herd immunity threshold in some populations could be as low as 20%^24^. In addition, there is some indication that immunity gained from infection by some common cold coronavirus strains may provide cross-immunity to SARS-COV-2^25,26^. There have also been reports that immunity gained from COVID-19 infection may wane, reducing individual and population levels of immunity over time. If these observations are indeed applicable here, the herd immunity threshold could be further modified^26^.

One important difference between immunisation by vaccination and by infection is that, during an epidemic, individuals with higher susceptibilities and/or larger numbers of contacts are likely to be infected earlier. If herd immunity is to be achieved by vaccination, optimal planning can reduce the coverage required to achieve herd immunity. For example, in an illustrative households model for variola minor infections in Brazil, it is shown that under the optimal vaccination strategy the proportion of the population that needs to be vaccinated is a third less than under a strategy that fully vaccinates randomly chosen households^27^. Although several COVID-19 vaccines have been developed, global demand in the early phases of vaccine roll-out still exceeds supply. Designing optimal vaccination strategies for different settings that take into account population structure alongside other public health concerns, e.g. protecting the vulnerable, could greatly enhance the chances of achieving herd immunity and the cost-effectiveness of vaccination as an intervention.

## How can R be estimated?

Before estimating R , the purpose of the estimation needs to be clarified. Is it intended simply to track the changes in the trajectory of case numbers over time? Or is it intended to assess the potential for pathogen transmission in a specific population, perhaps in the context of considering interventions? If the latter, the relevant population needs to be defined. Depending on the purpose, different data sets and statistical methods can be used.

There are several approaches to estimating $R_{t}$ from epidemiological data. In the most direct method, high-quality contact tracing data can be used, in theory at least, to estimate both $R_{t}$ and the generation time interval, and this has been attempted for COVID-19^28^. However, contact tracing of SARS-CoV-2 infections is notoriously difficult because of the high proportion of asymptomatic infections. Moreover, effective contact tracing reduces the number of contacts of traced individuals so that the corresponding estimates are biased.

More commonly, $R_{t}$ can be estimated by inferring the rate of infection transmission within a dynamical model fitted to observed cases, hospitalisations, deaths or a combination of those^29,30^. Dynamical models have been used widely to forecast the spread of COVID-19 and the effect of interventions. These models allow the impact of assumed changes in specific interventions on $R_{t}$ to be explored, so estimating $R_{t}$ in this way can be convenient. Dynamical models can be described by systems of differential equations and assume very large to infinite population sizes. In completely deterministic dynamical models, the uncertainty in estimated $R_{t}$ values depends only on data and parameter uncertainty, and not on stochastic uncertainty. However, if the number of new infections is small, the value of $R_{t}$ is strongly affected by chance events, which increases the uncertainty in the estimate. This situation can be addressed by the use of stochastic models or incorporating stochastic assumptions in otherwise deterministic model frameworks.

But this approach is not without drawbacks. Not least, $R_{t}$ estimates from dynamical models depend critically on assumptions (e.g. model structure and which parameter values are estimated), and on data quality. Another potential drawback is that many parameters of dynamical models are often assumed to be fixed over time. These approaches are therefore less suited to capture the effects of gradual, continuous changes in behaviour, mobility or social network structure. However, gradual changes in dynamic models can be incorporated by assuming that transmission parameters change over given intervals, while at the same time the possible amount of change is constrained to avoid big jumps caused by a small number of noisy data points^31^. In this way, models that include change-points in the rate of infection near specific interventions can infer the impact of control policies, as well as the effect of susceptible depletion.

There is also a difference in how $R_{t}$ is estimated between compartmental and agent- or individual-based models. In an agent-based model, it is possible simply to count exactly how many secondary infections are caused by each primary infection. Thus, all details of the epidemic including time-varying viral loads, population-level and localised immunity, interventions, network factors, and other effects are automatically incorporated and do not need to be considered separately^32^. As agent-based models explicitly include stochastic effects, the uncertainty in $R_{t}$ estimates can be greater than for those derived from deterministic dynamical models. Because of the greater number of parameters included in dynamical and particularly agent-based models, they require more data and more different types of data than the simpler statistical models described below to identify estimates for all parameters.

A third approach uses statistical models to estimate $R_{t}$ , and continuous changes in it, empirically from case notification data. These methods make minimal structural assumptions about epidemic dynamics, and only require users to specify the distribution of the generation interval. They are agnostic to population susceptibility or epidemic phase, but as we discuss below, care must still be taken to avoid quantitative and temporal biases. The most common empirical methods are the Cori method^33,34^ and the Wallinga–Teunis method^35^. The drawbacks of some statistical models include that they cannot be used to combine different data streams into a coherent picture.

Where genome sequences from viral samples taken from infected patients are available and the date of sampling is known, $R_{t}$ can also be estimated using phylogenetic methods. An evolutionary model is fitted that best explains the patterns of nucleotide substitution in the dated samples. The fitted model parameters include the nucleotide substitution rate and the population size of the virus at a given time in the past. Using a metapopulation analogy, the effective population size of a pathogen has been shown to be proportional to the number of infected individuals and inversely proportional to the transmission rate from which the reproduction number can be determined^36^.

### Statistical methods to estimate R

In this section, we discuss two frequently used simple statistical methods to estimate R and common issues associated with them. The Cori and Wallinga–Teunis methods estimate subtly different versions of $R_{t}$ ; the Cori method generates estimates of the instantaneous reproduction number and the Wallinga–Teunis method generates estimates of the case reproduction number^33,37^. The key difference is that the instantaneous reproduction number gives an average $R_{t}$ for a homogeneous population at a single point in time, whereas the case reproduction number can accommodate individual heterogeneity, but blurs over several dates of transmission. Furthermore, the case reproduction number is a leading estimator of the instantaneous reproduction number, i.e. it depends on data from after the time for which the reproduction number is to be estimated, and must be adjusted accurately to infer the impact of time-specific interventions^38^.

The instantaneous reproduction number represents the expected number of infections generated at time t by currently infectious individuals^33^. For real-time analysis, one of the benefits of estimating the instantaneous reproduction number is that it does not require information about future changes in transmissibility, and it reflects the effectiveness of control measures in place at time t . But as an aggregate measure of transmission by all individuals infected in the past (who may now be shedding virus), it does not easily consider heterogeneity in transmission. In contrast, the case reproduction number represents the expected number of infections generated by an individual who is first infected at time t and has yet to progress through the full course of viral shedding. This leads to ‘right censoring’ when the case reproduction number is estimated in real-time; if all infections generated by individuals who were infected at time t have not yet been observed, then the data must be adjusted^39–41^ or the case reproduction number will be underestimated.

The Cori method and the Wallinga–Teunis method involve inferring the values of $R_{t}$ that are most consistent with observed incidence data (for a review, see Gostic et al.^38^). In the Cori method, typically this inference is carried out by assuming that $R_{t}$ is constant over fixed time windows. Smoothing windows are used to avoid spurious fluctuations in estimates of $R_{t}$ . These can occur if imperfect observation and reporting effects, rather than actual bursts in transmission, are the main source of noise in the data. Cross-validation and proper scoring rules can be used to avoid under- or oversmoothing $R_{t}$ estimates^42^.

An important concept, basic to both methods, is the intrinsic generation time also referred to as the infectiousness profile. The intrinsic generation interval is a theoretical quantity derived from the renewal equation of Lotka and Euler^30,43^. It describes the time distribution of potentially infectious contacts made by an index case and is independent of population susceptibility^44^. In practice, the intrinsic generation interval is not observable, and it must be estimated carefully from observed serial intervals within contact tracing or household data^44–47^. The serial interval is generally defined as the duration of time between the onset of symptoms in an index case and in a secondary case^48^. In the early stages of an outbreak, accurate estimation should adjust for right truncation of observations, for changes over time in population susceptibility, and for interventions such as case isolation, which may shorten the generation interval by limiting transmission events late in the course of infectiousness^44,45,49^.

Both the Cori and Wallinga–Teunis methods are conceptually based on separating the infectiousness of an infective into two components, total amount and timing. The timing is expressed by the generation time distribution while the total amount is expressed by $R_{t}$ . The variation of (average) infectivity over time is ascribed, at least in practical implementations of the methods, to changes in $R_{t}$ , while the intrinsic generation time is assumed to remain fixed. This is a simplification that may lead to inaccurate estimation of $R_{t}$ , since, in reality, the observed generation time distribution varies over time, both because of the epidemic dynamics^48,50,51^, because of the epidemic affecting different subgroups of the population, with possibly different generation time distributions over time^52,53^, and, more importantly, because of interventions that affect the length or efficacy of the infectious period^49^. An additional complication is that the ‘intrinsic’ generation interval of the Cori and Wallinga–Teunis estimators includes potentially infectious contacts with both susceptible and immune individuals, whereas only contacts with susceptible individuals cause new infections, and are observed in contact tracing^44,45^. Even when using an accurately estimated fixed generation time distribution, both $R_{t}$ estimators are numerically sensitive to the specified mean and variance of the intrinsic generation interval^54^.

### Data used to estimate R

Fundamentally, $R_{t}$ is a measure of transmission. Ideally, it would be estimated from data on the total number of incident infections (i.e. transmission events) occurring each day. But in practice, only a small fraction of infections are observed, and notifications do not occur until days or weeks after the moment of infection. Temporally accurate $R_{t}$ estimation requires adjusting for lags to observation, which can be estimated as the sum of the incubation period and delays from symptom onset to case observation^54,55^. Delays not only shift observations into the future, but they also blur infections incident on a particular date across many dates of observation. This blurring can be particularly problematic when working with long and variable delays (e.g. from infection to death), and when $R_{t}$ is changing. Deconvolution^56–59^, or $R_{t}$ estimation models that include forward delays^60^ can be used to adjust lagged observations. Simpler approaches may be justifiable under some circumstances. If observation delays are relatively short and not highly variable, and if $R_{t}$ is not rapidly changing, simply shifting unadjusted $R_{t}$ estimates back in time by the mean delay can provide a reasonable approximation to the true value (see Challen *et al*.,^54^ in this volume, for an in-depth discussion). The advantages and disadvantages of each approach are reviewed in Gostic et al.^38^. Changes over time in case ascertainment can also bias $R_{t}$ estimates, so ideally data should be drawn from structured surveillance (see, e.g., the REACT study^61^) or adjusted for known changes in testing or reporting effort^61,62^.

In practice, $R_{t}$ can be estimated from a time series of new symptom onset reports, cases, hospitalisations or deaths. Choosing an appropriate data stream involves weighing representativeness, timeliness of reporting, consistency of ascertainment, and length of lag. For example, reported deaths may be reasonably unaffected by changes over time in ascertainment, but adjusting for long lags to observation can be challenging, and deaths may not be representative of overall transmission (e.g. if the epidemic shifts towards younger age groups)^63,64^. Extensions of existing statistical models for $R_{t}$ estimation could potentially integrate multiple kinds of data, by assuming that, for example, cases, hospitalisations and deaths, arise from a shared, latent infection process, with different delays^38^. A mechanistic model can also pull multiple data streams together by modelling the different processes underlying each data stream. Problems can arise if different data streams disagree on the progress of the pandemic. However, if the disagreement is caused by a shift in delays from events to reporting in different data streams, a mechanistic model can highlight these changes. Sometimes different data streams can be used for model validation.

All methods used to estimate $R_{t}$ must decide on the length of the time window over which it is to be estimated. All data used to estimate $R_{t}$ are noisy. The shorter the time window used for estimation, the higher will be the noise-to-signal ratio and, therefore, the uncertainty in the estimate of $R_{t}$ . In contrast, longer time windows will produce estimates with lower uncertainty, but sudden changes in transmission may not be detected if the time window is too long.

## Summary: cautions and recommendations

During the early phase of the epidemic:

-
$R_{0}$ estimates in the early phase may not be representative for the population as a whole if the group of initial transmitters is atypical.
-
$R_{0}$ may be incorrectly estimated in the early phase if infected but asymptomatic individuals are not counted or recognised, and their epidemiologically relevant behaviour differs from that of symptomatic individuals.

When the epidemic is established in the population:

-
$R_{t}$ can differ for different population groups, and the value of $R_{t}$ is dominated by the group in which most transmission occurs. To improve targeted containment measures, where possible additional information should be reported alongside case data, such as demographic, socio-economic and occupational information.
- The estimated value of $R_{t}$ and its associated uncertainty depend on the data stream(s) used and the time window over which $R_{t}$ was estimated, and these should be reported alongside the estimates. This will make it possible to draw more robust conclusions when considering results from different models.
- Model components that are likely to change over the time course of the epidemic (e.g. the generation time distribution) should be updated regularly, and sensitivity to changing assumptions should be kept under consideration.

When the ongoing epidemic is fragmented:

-
$R_{t}$ estimates from local outbreaks, if they can be contained, cannot inform on the progress of the epidemic and efficacy of interventions at the national level. They may inform local interventions. Other descriptors should be considered to assess the progress of the epidemic, such as the number of new cases per capita per day in a defined area, the number of hospitalisations and the spare hospital and intensive care capacity.
- Imported cases that are effectively quarantined should not be counted towards $R_{t}$ estimates as they do not contribute to the local transmission potential in the community.

Vaccination and herd immunity:

- If the available vaccine supply is limited, optimal vaccination strategies should be designed that take into account population structure and the transmission potential within different groups and other public health priorities, e.g. protection of the vulnerable groups.

In conclusion, estimated R values do not exactly correspond to the theoretically defined quantities. In statistical terms, model uncertainty, sampling variability, and data accuracy affect the estimates. Nevertheless, $R_{0}$ and $R_{t}$ are useful quantities to assess the potential and progress of an epidemic. Their usefulness for decision making varies depending on the phase of the epidemic (early, established and fragmented). Clearly defining the context, the data streams and the statistical methods used to estimate R can improve its value for the management of an epidemic.
